# Supplementary material for: A Comprehensive Assessment of Ultraviolet-Radiation-Induced Mutations in Flammulina filiformis Using Whole-Genome Resequencing
Source: J Fungi (Basel). 2024 Mar 20;10(3):228. doi: 10.3390/jof10030228 (PMC10971301; doi:10.3390/jof10030228)
Supplement: Supplementary file 1 [file jof-10-00228-s001.zip › Supplementary Material S8/KEGG annotation/out/64381550635650.os/KO/out_map/map05131.html]

KEGG PATHWAY: Shigellosis - Reference pathway


|  |  |
| --- | --- |
| **Shigellosis - Reference pathway** |  |

[
Pathway menu
| Organism menu
| Pathway entry
| Show description
| User data mapping
]

Shigellosis, or bacillary dysentery, is an intestinal infection caused by Shigella, a genus of enterobacteria. Shigella are potential food-borne pathogens that are capable of colonizing the intestinal epithelium by exploiting epithelial-cell functions and circumventing the host innate immune response. During basolateral entry into the host-cell cytoplasm, Shigella deliver a subset of effectors into the host cells through the type III secretion system. The effectors induce membrane ruffling through the stimulation of the Rac1-WAVE-Arp2/3 pathway, enabling bacterial entry into the epithelial cells. During multiplication within the cells, Shigella secrete another subset of effectors. VirG induces actin polymerization at one pole of the bacteria, allowing the bacteria to spread intracellularly and to infect adjacent cells. OspF, OspG and IpaH(9.8) downregulate the production of proinflammatory cytokines such as IL-8, helping bacteria circumvent the innate immune response.

|  |  |
| --- | --- |
| Reference pathway | 100% |
